# Benefits of combined quantitative and qualitative evaluation of learning experience in a gerodontology course for dental students

**DOI:** 10.1186/s12909-020-02196-0

**Published:** 2020-08-26

**Authors:** Ragna Lamprecht, Jennifer Guse, Martin Schimmel, Frauke Müller, Guido Heydecke, Daniel R. Reissmann

**Affiliations:** 1 Department of Prosthetic Dentistry, Center for Dental and Oral Medicine, University Medical Center Hamburg-Eppendorf, Martinistrasse 52, 20246 Hamburg, Germany; 2 Department of Medical Psychology, University Medical Center Hamburg-Eppendorf, Hamburg, Germany; 3 Department of Reconstructive Dentistry and Gerodontology, University of Bern, Bern, Switzerland; 4 Division of Gerodontology and Removable Prosthodontics, University of Geneva, Geneva, Switzerland

**Keywords:** Gerodontology, Education, Teaching, Dental students, Quantitative evaluation, Qualitative evaluation

## Abstract

**Objectives:**

The oral health status of long-term care (LTC) facility residents is often poor, and acceptance of dental services by the elderly is irregular and mostly problem-driven. The perceived knowledge gap due to insufficient under- or postgraduate education and training in gerodontology might present a barrier for dentists to provide domiciliary care. This study aimed to develop a high-quality student course in gerodontology.

**Methods:**

A total of 52 undergraduate dental students (age: 23.4 ± 2.1 yrs., 81% female) participated in a novel one-year gerodontology course and were included in this prospective study. The course was organized over two semesters, comprising two consecutive modules (one theoretical and one practical). The evaluation after the first semester applied a 16-item questionnaire with an ordinal 6-point response scale ranging from “not satisfied at all” (0) to “very satisfied” (5) for quantitative evaluation, and free-text comments as the qualitative part. These qualitative findings were used for validating the satisfaction questionnaire by triangulation, and to identify potential issues for improving the course. Satisfaction scores of the second evaluation after 1 year were used to assess potential effects of course modifications by comparing the participant satisfaction scores between both evaluations.

**Results:**

Satisfaction scores of 3.6 ± 0.7 after the first semester indicated students’ initial satisfaction. The lowest satisfaction was observed for timeframe (2.6 ± 1.3) and interdisciplinary education (3.0 ± 1.4). The qualitative evaluation confirmed not only the ratings but also provided potential explanations, which were addressed by modifying the course accordingly. The effect of the modifications on participant evaluation was reflected by substantially improved satisfaction scores at the second assessment in 14 of 16 items, with a significant increase in overall satisfaction from 3.6 ± 0.7 to 4.0 ± 0.4 (*p* = 0.008).

**Conclusion:**

A combined quantitative and qualitative evaluation is a successful method for developing, evaluating, and improving a gerodontology course for dental students with high student satisfaction.

## Introduction

Aging of the general population is a nearly global phenomenon and particularly evident in high-income countries. This demographic shift leads to a growing number of people worldwide aged 60 years and older; this population is expected to reach two billion individuals by 2050 [1]. The elderly are more frequently affected by chronic diseases, with approximately 50% presenting with at least two chronic conditions such as cardiovascular disease, hypertension, or diabetes mellitus, which can lead to disability or death [2]. The prevalence of severe cognitive and physical disabilities increases drastically with age [1]. Multimorbidity is accompanied by reduced functional capacity and self-reliance, leading to complex care needs. Accordingly, many elderly individuals are care-dependent; in Germany alone, 3.4 million persons depend on care, of whom approximately 818,000 live in long-term care (LTC) facilities [3].

In LTC facility residents, oral health status is often poor, with a higher prevalence of caries (24.5 vs. 21.6 DMF-T index), fewer natural teeth (22.4 vs. 17.8 missing teeth), and an increased number of untreated caries lesions than among non-dependent elderly individuals [4]. While one-third of the 75–100 – year-old demographic has no natural teeth, every second dependent elderly individuals is edentulous. As a result of increasing care dependency, the ability of self-determined activities is reduced. Thus, a substantial number of LTC facility residents (29.8%) require assistance performing daily oral hygiene measures relative to non-dependent elders (6.7%) [4]. Accordingly, LTC facility residents are more often affected by deteriorated oral health as well as functional and psychosocial impairments [5]. Furthermore, a low number of natural teeth is a significant risk factor for malnutrition. Poor chewing function affects the diet by limiting the type and amount of food intake. This can have negative effects on general health, increase risks for various noncommunicable diseases, and reduce overall quality of life [6, 7]. Despite the high need for dental care to the care-dependent elderly, many are not regularly seen by a dentist and are undersupplied in terms of dental care [8, 9]. The logistic effort, time, and cost of dental treatments in LTC facilities is often considered not profitable by many dentists. Furthermore, the confrontation with aging and death may present an emotional burden, hindering dentists from providing domiciliary oral health care to LTC facility residents [10]. Another reason might be a lack of under- and postgraduate education and training in gerodontology.

In Europe, dental education in gerodontology has received a higher priority over recent decades. One study indicated that in 86.2% of 123 European dental schools, gerodontology was taught at the undergraduate level [11]. In contrast, only 62.5% of 56 US dental schools indicated teaching gerodontology as an independent course [12]. Other countries, such as Brazil and India, have only recently started to establish gerodontology education in their dental curricula [13, 14]. Even though most dental schools in Europe teach gerodontology, the content varies substantially and it is not clear whether freshly graduated dentists are well-prepared for providing oral health care to the elderly [11, 15]. In 2009, the European College of Gerodontology (ECG) published undergraduate curriculum guidelines in gerodontology [16] in order to standardize education and training across European dental schools. According to these guidelines, it is necessary to develop competencies in dental service for the elderly to qualify dental professionals for future challenges concerning the aging population. The ECG guidelines recommend interdisciplinary and interprofessional training. They emphasize a vertical integration of contents related to gerodontology throughout the curriculum, with theoretical and practical training in preclinical and clinical students. However, most dental school gerodontology training programs teach only little theoretical content matrixed within general dental studies. Clinical experience with older patients is limited and mostly offered in a dental school environment, and not in outreach locations, particularly LTC facilities [17]. The majority of gerodontology education is neither integrated with medical disciplines nor taught in an interdisciplinary and interprofessional team approach. Furthermore, education is rarely offered for both undergraduate and postgraduate students [11]. Hence, most of the current courses in gerodontology worldwide do not meet the standards recommended by the ECG. They do not include practical training in LTC facilities and do not provide dental care for the residents. Furthermore, programs are often solely evaluated quantitatively and instruments for evaluation were developed without including target population, i.e. dental students.

The aim of this study was to develop a high-quality undergraduate course in gerodontology while making use of a combined quantitative and qualitative evaluation to identify and address potential opportunities to improve students’ learning experience.

## Methods

### Study design, setting, and subjects

In this prospective study, a total of 52 dental students (23.4 ± 2.1 yrs., 81% female) at the University of Hamburg, Germany, voluntarily participated in the gerodontology course. The only inclusion criterion for the students was that they were in the second, third, or fourth year of their undergraduate dental curriculum. Duration of undergraduate dental program is usually 5 years, followed by state exam. During first 3 years also referred to as pre-clinical part of dental studies students acquire practical skills by practicing on phantoms, whereas in the last 2 years in the clinical part students practice on real dental patients, i.e. they perform regular dental treatments. Therefore, year 2 students usually do not have any patient contact. Year 3 students already perform some oral examinations among themselves and assisted their fellow students in higher years. That is, they have some clinical experience but did not examine or treat real patients. In contrast, year 4 students care for real dental patients, what is comparable to what normal dentist do, but of course students are supervised by lecturers. Two introductory lectures on gerodontology are given on a regular basis at the end of year four. Accordingly, year 3 students are prepared and year 4 students somewhat experienced in dental patient care, but theoretical knowledge in gerodontology is basically identical within participating students of different years.

The study was designed for a period of 1 year, during which the gerodontology course was evaluated twice: once after 6 months and again at the end of the second unit after 1 year. Findings from the intermediate evaluation were used to address students’ concerns and suggestions for improvements, and to modify the course accordingly.

The study was carried out in accordance with the Declaration of Helsinki. The study protocol was approved by the Dean of the University Medical Center Hamburg-Eppendorf, Hamburg, Germany since the research protocol was not deemed to be biomedical or epidemiological research. Participation in the study was voluntary. There was no disadvantage to those who chose not to participate. Participants were able to opt-out of the study without experiencing disadvantages.

### The gerodontology course

The gerodontology course was developed as a one-year course comprised of two six-month (semester) units. The two consecutive units each comprised of two modules, one theoretical and one practical one. The course was conceived and run by the Department of Prosthodontic Dentistry in cooperation with the Department of Primary Care of the University Medical Center Hamburg-Eppendorf, Germany, as well as in three nursing homes.

The theoretical module of the course consisted of three didactical formats: lectures, seminars, and case-related presentations. At the beginning, students were provided lectures with basic information on epidemiology, economic, and social issues of aging, on physiological and pathological changes associated with age, and on geriatric assessment [18]. Subsequent seminars provided in-depth knowledge regarding general health and medical care, and examination principles for elderly LTC residents. Students were instructed how to manage and treat patients in nursing homes, providing basic and essential competencies to successfully perform the subsequent hands-on module.

In the practical module, students were allocated to groups of three or four. Each group was supervised in the nursing homes by two dentists, and visited their assigned residents twice per semester in their LTC facilities. During the first visit, students recorded the medical history and performed a geriatric assessment, including assessments on nutrition, oral health, and a psychosocial screening. This was complemented by a comprehensive clinical oral examination. Based on the collected information, students developed together with their tutor an individual treatment plan for their patients, which was presented and discussed with the group during subsequent case review sessions. During the second visit, the coordinated treatment plan was applied after obtaining informed consent from the patient, and necessary minor dental treatments were performed using a portable treatment unit. Students cleaned and restored teeth and prostheses and gave practical advice on oral hygiene to improve oral health. This was complemented by nutritional advice in order to improve the residents’ nutritional state with a long-term perspective. In general, dental care comprised of acute pain therapy, conservative treatment, simple restorations and denture repairs, and maintenance care.

Year 2 students performed anamnesis and checked medical history. Oral exams were performed by year 3 students and treatments basically by year 4 students. However, since at least one student of each years was in a team, even year 2 students could assist oral exam and dental treatment. Students of different years completed the course simultaneously according to the multi-semester approach.

Differences in knowledge and clinical competence of participating students was part of our concept, i.e. the multi-semester approach. In the practical training, teams consisted of students of different levels of clinical competences. That is, students with high clinical competence instructed and supported students with low or no clinical competence. This multi-semester approach was based on the theory of students teach students, supervised by lecturers. In lectures and seminars, information provision was tailored according to students’ level of knowledge. That is, theoretical knowledge on gerodontology, e.g. information on ageing, geriatric assessment, medical care and common diseases of the elderly, and treatment options and strategies in geriatric oral health care, was provided to all participants since level of knowledge was expected to be identical, i.e. all students had insufficient knowledge on this topic. In contrast, specific seminars especially for preparation for patient contact and examination were provided for year 2 and year 3 students. This should ensure all participants were well prepared for being confronted with aging and specific oral health care needs in the elderly.

### Assessment of student satisfaction

For evaluating the newly implemented gerodontology course, a combined quantitative and qualitative evaluation was applied, consisting of a 16-item questionnaire on participant satisfaction for quantitative assessment and free-text comments as the qualitative element. This assessment was designed to identify potential for improving the course.

The questionnaire was developed based on methodology suggested by *Kirkpatrick & Kirkpatrick* [19], and contains a four-level evaluation model for evaluating training programs. The four levels are: reaction, learning, behavior, and results. It helps to objectively analyze and improve the training. First, in workshops with the students and lecturers, relevant topics regarding students’ expectations and objectives of the evaluation were identified. The results of these workshops formed the basis for a 20-item preliminary version of questionnaire. Subsequently, lecturers reviewed the items to verify content was unambiguous and easy to comprehend until consensus was reached. To reduce the number of items, lower respondent burden, and identify variables most relevant to course participants, 31 of the students in the gerodontology course anonymously rated the importance of each item with respect to students’ expectations on the course using on a 4-point ordinal rating scale ranging from 1-“unimportant” to 4-“very important”. Items with ratings of 1 or 2 (“less important”) were eliminated. The final questionnaire comprised 16 items with statements relating to the topics *reaction* and *learning* (Table 1), and using a 6-point Likert scale ranging from 0-“strongly disagree” to 5-“strongly agree”.

**Table 1:** Correlation between item and subdomain scores

#	Item	Reaction			Learning	
		Organization	Psychosocial aspects	Cooperation	Theoretical knowledge	Psychosocial competence
		*Pearson correlation coefficient*				
1	Timeframe	**0.72**	0.20	0.24	0.50	0.46
2	Emotional overload	0.26	**0.77**	0.39	0.15	0.24
3	Implementation option	**0.81**	0.21	0.49	0.38	0.37
4	Supervision	**0.77**	0.31	0.38	0.33	0.21
5	Cooperative collaboration	0.38	0.56	**0.66**	0.24	0.27
6	Human experience	0.16	**0.61**	0.37	0.49	0.55
7	Semester across collaboration	0.26	0.31	**0.67**	0.53	0.46
8	Interdisciplinary education	0.40	0.31	**0.83**	0.62	0.40
9	Knowledge acquisition on elderly	0.40	0.50	0.46	**0.69**	0.63
10	Sensitization	0.33	0.50	0.29	0.69	**0.75**
11	Preparation for professional life^a^	0.41	0.26	0.22	0.39	0.54
12	Increase communication skills	0.39	0.46	0.53	0.50	**0.80**
13	Knowledge acquisition on systemic diseases and oral health	0.45	0.22	0.53	**0.86**	0.56
14	Knowledge acquisition on treatment options	0.44	0.35	0.56	**0.86**	0.57
15	Social competencies	0.37	0.29	0.41	0.51	**0.80**
16	General satisfaction^a^	0.62	0.33	0.47	0.72	0.73

Items in bold are components of the corresponding subdomain score

^a^ Items not included in subdomain scores

Internal consistency within the questionnaire was determined for the first assessment using Cronbach’s alpha [20] and average inter-item correlation. Alpha values were interpreted according to guidelines, with values of 0.70 or above suggesting satisfactory reliability [21]. For broad higher order constructs, such as satisfaction with several aspects of educational courses, an average inter-item correlation of at least 0.15 is considered satisfactory [22]. Cronbach’s alpha (0.89) and average inter-item correlation (0.33) revealed that the instrument’s internal consistency was excellent.

Based on item content, we defined subdomains for the topics reaction and learning and allocated the items to the appropriate subdomain (Table 1). Only item 11 (*Preparation for professional life*) and item 16 (*General satisfaction*) were not assigned to a specific subdomain since they represent overall evaluations. The subdomain scores were analyzed as the mean of the contributing items. Pearson correlation analyses were performed using the data from the first assessment to estimate the strength of the correlation between the subdomain scores with the 16 single items, to test whether the item scores correlate with the appropriate subdomain score. As expected, all items correlated higher with their corresponding subdomain score than with other subscale scores, indicating a correct allocation of the items to the topics (Table 1).

For the qualitative evaluation, students were prompted to provide free-text comments on three topics: *praise*, *criticism*, and *suggestions for improvement*. There were no restrictions in terms of length or quantity of the comments.

### Analyses

The first part of the analysis in this study comprised three steps. First, findings from the quantitative assessment of student satisfaction were evaluated by calculation of means and standard deviations (SD) of the students’ ratings. Additionally, item scores were dichotomized using the values 3–5 (positive half of the scale) to indicate agreement (satisfaction) and the values 0–2 (negative half of the scale) for non-agreement (dissatisfaction). Second, free-text responses were analyzed by conventional content analysis with inductive categorization according to *Hsieh* et al. [23]. Responses were read repeatedly to gain a sense of the whole. Subsequently, two authors (RL, JG) reviewed the responses word by word to identify key concepts and generate code labels. These codes were allocated to categories, which were reviewed by all authors until a consensus was reached. These categories represent all relevant topics mentioned by the students regarding praise, criticism, and suggestions for improvement. Finally, by means of triangulation, the findings of the quantitative and the qualitative evaluation to validate the questionnaire for student satisfaction with the free-text comments were compared. These three steps of the analysis were performed for both evaluations, after the first semester and at then the end of the gerodontology course after two semesters. The first evaluation was primarily used to identify problems and identify any potential for improvement. Based on the topics raised, the course was modified and adjusted.

The second part of the analysis involved comparing the two qualitative evaluations. We hypothesized that changes in questionnaire scores were in part due to modifications and adjustments introduced in the second stage of the gerodontology course. The survey’s summary and subdomain scores were compared using t-tests for independent samples, since the anonymous completed questionnaires could not be allocated individual students. Furthermore, standardized effect sizes (Cohen’s d) were calculated to determine the strength of change between the two assessments. Effect size represents the degree of difference and is defined as difference between means divided by the pooled SDs of measures. Guidelines suggest that an effect size of 0.2 is considered small, 0.5 medium, and 0.8 large [24]. Additionally, the magnitude of the effect size was compared with the value, defining a minimally important difference in self-reported scores (0.5), as determined by literature review [25].

All statistical analyses were performed using the statistical software package STATA/MP (Stata Statistical Software: Release 14.2. StataCorp LP, College Station, TX), with the probability threshold of a type I error set at 0.05. Qualitative data was analyzed using MAXQDA (Release 10. VERBI Software GmbH, Berlin, Germany).

## Results

### Student characteristics

After the first semester, 48 of 52 participating students (response rate: 92.3%) evaluated the gerodontology course (Table 2). Their mean age was 23.4 ± 2.1 years and 81.3% of the students were female. Most of the students (79.2%) were in the third and fourth year of their dental studies.

**Table 2:** Characteristics of participating students during first and second assessment

	1. Assessment	2. Assessment	
	*n* = 48	*n* = 35	Significance
	*Mean (SD) [range] or n (%)*		*P-value*
**Age**			
Years	23.4 (2.1) [21; 29]	24.1 (2.2) [21; 30]	0.151
**Gender**			
Male	9 (18.8)	6 (17.1)	0.851
Female	39 (81.3)	29 (82.9)	
**Semester**			
Fourth	10 (20.8)		0.734^a^
Fifth		4 (11.4)	
Sixth	24 (50.0)		
Seventh		22 (62.9)	
Eighth	14 (29.2)		
Ninth		9 (25.7)	

^a^ Based on semester when entering the study

At the second assessment, 35 evaluations of 50 participating students (response rate: 70.0%) were available for analyses (age: 24.1 ± 2.2 years; proportion of female students: 82.9%). The number of students being in the clinical part of their dental studies (88.6%) did not change significantly (all *p* > 0.05).

### Initial evaluation

#### Satisfaction scores

At initial evaluation, i.e. after one semester, students rated the course mainly positively with item means ranging from 2.6 for *time frame* to 4.4 for *emotional overload* (Table 3). The average of all items was 3.6, corresponding to 89.8% overall satisfaction (Table 4).

**Table 3:** Item scores for both assessments

#	Item	1. Assessment			2. Assessment		
		*Mean (SD)*	*Range*	*%*	*Mean (SD)*	*Range*	*%*
1	Time frame	2.6 (1.3)	[0; 5]	55.1	4.1 (1.0)	[2; 5]	91.4
2	Emotional overload	4.4 (1.1)	[0; 5]	93.9	4.8 (0.4)	[4; 5]	100.0
3	Implementation option	3.1 (1.4)	[0; 5]	71.4	3.3 (1.3)	[0; 5]	80.0
4	Supervision	4.1 (1.0)	[1; 5]	91.8	4.5 (0.7)	[2; 5]	97.1
5	Cooperative collaboration	3.8 (1.3)	[0; 5]	85.7	3.5 (1.3)	[1; 5]	70.6
6	Human experience	4.2 (0.9)	[1; 5]	93.9	4.5 (0.7)	[3; 5]	100.0
7	Semester across collaboration	3.9 (1.3)	[1; 5]	85.7	4.1 (1.0)	[2; 5]	91.4
8	Interdisciplinary education	3.0 (1.4)	[0; 5]	65.3	3.5 (1.4)	[0; 5]	79.4
9	Knowledge acquisition on elderly	3.8 (1.0)	[1; 5]	91.8	4.2 (0.8)	[2; 5]	94.3
10	Sensitization	3.9 (1.2)	[0; 5]	87.8	4.4 (0.8)	[2; 5]	94.3
11	Preparation for professional life	3.1 (1.5)	[0; 5]	71.4	3.3 (1.2)	[1; 5]	77.1
12	Increase communication skills	3.6 (1.1)	[1; 5]	83.7	4.1 (0.8)	[2; 5]	97.1
13	Knowledge acquisition on systemic diseases and oral health	3.6 (1.1)	[0; 5]	83.7	3.5 (1.2)	[0; 5]	88.6
14	Knowledge acquisition on treatment options	3.6 (1.1)	[1; 5]	79.6	3.7 (1.0)	[1; 5]	88.6
15	Social competencies	3.5 (1.1)	[1; 5]	87.8	4.0 (0.7)	[2; 5]	97.1
16	General satisfaction	3.5 (1.3)	[0; 5]	79.6	4.2 (0.6)	[3; 5]	100.0

**Table 4:** Domain and subdomain scores for both assessments, and statistical significance and size of change between assessments

Domains and subdomains	1. Assessment		2. Assessment		Change	
	*Mean (SD)*	*%*	*Mean (SD)*	*%*	*P-value*	*ES*
**Reaction**	3.6 (0.7)	87.8	4.0 (0.5)	100.0	0.004	0.66
Organization	3.3 (0.9)	79.6	3.9 (0.7)	97.1	0.001	0.79
Psychosocial aspects	4.3 (0.7)	98.0	4.6 (0.4)	100.0	0.013	0.56
Cooperation	3.6 (1.0)	87.8	3.7 (0.7)	94.3	0.474	0.16
**Learning**	3.6 (0.8)	87.8	3.9 (0.5)	100.0	0.055	0.43
Theoretical knowledge	3.7 (0.9)	91.8	3.8 (0.7)	97.1	0.391	0.19
Psychosocial competence	3.7 (0.9)	89.8	4.2 (0.5)	100.0	0.003	0.69
**Total**	3.6 (0.7)	89.8	4.0 (0.4)	100.0	0.008	0.60

When considering domain scores, high satisfaction was observed for the domains *reaction* (87.8%, mean 3.6) and *learning* (87.8%, mean: 3.6; Table 4). Among the subdomains, the highest satisfaction was indicated for *psychosocial aspect* (98%; mean 4.3). However, satisfaction was substantially lower for subdomains *cooperation* (87.8%, mean 3.6) and *organization* (79.6%, mean 3.3), suggesting potential for further improvements to the course.

#### Qualitative comments

A total of 111 qualitative comments on the gerodontology course (praise: *n* = 42; criticism: *n* = 32; suggestions for improvement: *n* = 37) were provided in the evaluation after the first semester (Table 5). They were analyzed and used to generate codes, which were subsequently sorted into four themes: *idea of the course*, *organization and structure*, *supervision*, and *learning experience*.

**Table 5:** Representative examples of categorized free text responses from students

Categories and codes	Praise (*n* = 42)	Criticism (*n* = 32)	Suggestions (*n* = 37)
**Idea of the course**			
Nursing home visits	*“The visit to the nursing home was very good.”*		
Concept of the course	*“I think the idea of the project is very good and important.”*		
Good supplement to common dentistry studies	*“A good complement to the everyday university life.”*		
**Organization and structure**			
Multi-semester approach	*“Working for more than one semester was very exciting.”*	*“It’s difficult to combine the fourth, sixth and eighth semester in one course.”*	
eLearning resource	*“The group at Moodle is well structured.”*		
Timeframe		*“Unfortunately, it’s not possible to combine it with all timetables because of the time.”*	*“The weekly lectures should rather be bi- or tri-weekly, and can then also be longer”*
Nursing home visits		*“Unfortunately, the implementation (medical treatment,* etc.*) in the nursing homes is very limited.”*	*“More practical training!”*
Courses	*“Good organization!”*	*“Fewer lectures - instead teach more regarding oral health in old age!”*	*“Fewer lectures, more seminars for preparation for the nursing home visits!”*
**Supervision**			
Faculty motivation	*“The lecturers were motivated; they also explained many things in the nursing home.”*		
Faculty support and supervising	*“I always felt very well supervised.”*		
Accessibility	*“The tutors were always accessible.”*		
**Learning experience**			
Theoretical knowledge	*“The training course of the foundation was great!”*	*“Too little new knowledge gained from the lectures.”*	*“Fewer lectures, more seminars!”*
Practical training	*“Good handling with the patients; you gain experience how difficult it can be to treat patients.”*	*“Because of being six persons per patient, it wasn’t possible to learn much, to question the patient, nor to establish a relationship.”*	*“More preparation for the nursing home visits,* e.g. *a role-playing game.”*
Experience with older patients	*“The project itself is great - and the patients were mostly very pleased.”*		
Psychosocial skills	*“A good complement to the everyday university life, especially supporting the social and communicative characteristics as well as working with more than one semester, melded together.”*		

For the category *idea of the course*, the qualitative evaluation revealed high satisfaction, especially for nursing home visits (*“The visit to the nursing home was very good”),* the concept of the course (*“I think the idea of the project is very good and important”),* and being a good supplement to the regular dental curriculum (*“A good complement to the everyday university life”)*.

Aspects belonging to the category *organization and structure* were also positively evaluated; especially the multi-semester approach (*“Working for more than one semester was very exciting”)* was emphasized. Most of the critical comments for this category were related to the time frame of the course in general (“*Too time-consuming”*). In particular for the practical module, the space of time for each patient and the implementation (medical treatment, etc.) were limited (*“Unfortunately, the implementation (medical treatment, etc.) in the nursing homes is very limited”).* Suggestions were provided with respect to further optimization of the time frame (*“Fewer lectures - instead teach more regarding oral health in old age!”).* It was generally recommended to increase the total course length in order to better prepare participants for the first patient examinations. Additionally, the number of nursing home visits was suggested to be increased *(“More nursing home visits would be great!”).*

For the category *supervision*, especially *faculty motivation (“The lecturers were motivated; they also explained many things in the nursing home”), support*, and *supervising* (*“I always felt very well supervised”)* were praised by students. There was no criticism and no suggestions for improvement in this category.

Finally, aspects of the category *learning experience*, mainly in the practical module, were rated as very good (*“Good handling with the patients; you gain experience how difficult it can be to treat patients”).* Moreover, students were satisfied with the experience of older patients during the practical training (*„The project itself is great - and the patients were mostly very pleased”).* Nevertheless, students suggested supporting the preliminary training with an increased number of seminars before visiting the nursing homes (*“More preparation for the nursing home visits, e.g. a role-playing game”)*. The theoretical training was somewhat criticised due to high frequency and too little knowledge gained by students in their fourth year (*“Too little new knowledge gained from the lectures”*). Students suggested preparing specific lecture and seminar content according to the foreknowledge of the students, i.e. to provide individual classes for the students from different years of study. In addition, students recommended a reduction in the number of lectures and an increase in the number of seminars (*“Fewer lectures, more seminars!”*).

#### Triangulation

Qualitative comments also reflected the quantitative satisfaction scores. That is, high mean satisfaction scores corresponded well with the mainly positive free-text comments. The categories *idea of the project* and *supervision* were rated positively in the qualitative evaluation. Accordingly, the items *semester across collaboration* and *supervision* had high satisfaction scores. Many free-text comments praised the learning experience at the nursing home visits, which is reflected in high satisfaction scores of several items such as *knowledge acquisition on elderly*, *human experience*, and *social competencies*.

Satisfaction for the subdomains *organization* and *cooperation* were only moderate, especially for the items *time frame* (55.1%, mean 2.6) and *interdisciplinary education* (65.3%, mean 3.0). The free-text comments for the category *organization and structure*, especially *time frame*, revealed criticism and suggestions for improvement.

Overall, agreement between qualitative comments and satisfaction scores indicated sufficient validity of the newly developed satisfaction questionnaire.

### Final evaluation

At the final evaluation after 1 year, mean item scores ranged from 3.3 for *implantation option* and *preparation for professional life* to 4.8 for *emotional overload* (Table 3). The overall score of pooled items was 4.0, corresponding to 100% satisfaction (Table 4).

When comparing both quantitative assessments, student satisfaction substantially improved from the first to second semester, indicated by higher satisfaction scores in 14 out of the 16 items at second assessment (Table 3), with a statistically significant (*p* = 0.008) and clinically relevant (ES = 0.60) improvement in the total satisfaction score (Table 4). Higher satisfaction after implementation of course modifications for the second semester was also reflected in the subdomains *organization* (*p* = 0.001, ES = 0.79), *psychosocial aspects* (*p* = 0.013, ES = 0.56), and *psychosocial competence* (*p* = 0.003, ES = 0.69).

## Discussion

This is the first study applying sophisticated methodology in developing an evaluation instrument, practicing a multi-semester and interdisciplinary approach with significant practical training, and demonstrating feasibility and benefit of a combined quantitative and qualitative evaluation for developing and continuously improving a gerodontology course for dental students. Even though students mostly rated the newly developed and implemented gerodontology course positively in a mid-course evaluation, also reflected in the qualitative evaluation, the students’ satisfaction also significantly increased in a course-end evaluation after addressing critical comments and appraisals by modifying the course, especially for issues raised during the first evaluation.

Our findings suggest that a gerodontology course with a multi-semester approach can be successfully implemented in the dental curriculum and is well received by the students. Especially for the practical training, teaming of students from different years was very effective for their learning experience. They learned from one another and enhanced their knowledge. In comparison, semester collaboration was not effective for the theoretical education, mainly because the level of knowledge varied strongly among students. Students closer to receiving their degree benefited less from theoretical courses and seminars. Consequently, the latter required individual adaptation to the various levels of knowledge. Furthermore, the relationship between practical training and theoretical education was criticized, with several suggestions for improvement. After extending nursing home visits, ratings of learning experience and satisfaction increased significantly. In addition, some lectures were replaced by small group seminars, since these were considered essential for theoretical preparation and prevention of emotional overload. Finally, gerodontology as a subfield cannot solely focus on dentistry, necessitating the involvement of other medical disciplines. Therefore, a cooperation with the Department of Primary Care was established to strengthen the interdisciplinary character of the course and cover as many aspects of oral health care for the elderly as possible. After modification, satisfaction scores increased, indicating that the implemented modifications were highly effective in addressing students’ concerns, indeed leaving little room for further improvement, according to the free-text comments.

With respect to the methodological approach, the findings from the qualitative evaluation were very beneficial for validating the quantitative segment of the satisfaction questionnaire and identifying potential improvements. By triangulation, findings from the qualitative analysis could be compared to the scores of the satisfaction questionnaire’s individual quantitative items. The pattern of high and low satisfaction scores was consistently reflected in the free-text comments. The content of topics rated as “highly satisfied” were associated with mainly positive comments, whereas for items with lower quantitative satisfaction, most comments were critical and several suggestions for improvements were provided. No free-text comment addressed any issue not covered by the satisfaction questionnaire. Hence, the development of the questionnaire using workshops with focus groups and involving the target population, i.e. dental students, ensured sufficient construct and content validity of the questionnaire. Another important aspect of the qualitative evaluation that should not be underestimated was the provision of specific comments regarding potential improvements to the course. While a quantitative evaluation highly standardizes student satisfaction in a manner that the obtained scores can be compared across studies, identifying explanations for low satisfaction and developing tailored modifications can be very challenging. For instance, in our study the item *timeframe* and the domain *organization* received the lowest satisfaction ratings in the first evaluation. Using the categorized free-text comments, interventions addressing these issues could be identified (e.g. *“The weekly lectures should rather be bi- or tri-weekly, and can then also be longer”;* Table 5).

The positive effects observed here and the need to implement gerodontology training in dental studies confirm results from previous studies [26, 27]. All reported gerodontology courses have similar theoretical content, in Europe mostly in agreement with the ECG. However, the schedule during the dental curriculum when the gerodontology training was offered and the course duration varied. Moreover, practical training is not established as standard in all countries, e.g. in Germany only a few universities offer both theoretical education and practical training [28]. Courses were evaluated differentially with respect to the methodical approach. In the Zürich and Leipzig programs, students completed a questionnaire with ten items and were offered a choice of positive statements to characterize the course [29]. In particular, the practical training with MobiDent in Zürich was rated very positively. In Leipzig, high frustration was reported due to having no facilities for dental treatment of the elderly, which is indicated in lower satisfaction ratings of *mental strain* compared to high ratings of *emotional overload* in our study. In a pilot study conducted in Athens, students evaluated a questionnaire compared with open-ended questions [30]. In comparison to our study, general impression of the course had lower ratings than *general satisfaction* in our study. One potential explanation for this is that we utilized qualitative comments for improving the course and not only for course evaluation as was done in the Athens; however, cultural differences between countries may also apply. In Japan, an eight-day course with a problem-based learning tutorial was introduced [31]. Students’ theoretical knowledge and perceived competencies in gerodontology were evaluated before and after the course. Unlike the present study, students only examined nursing home residents and developed treatment plans without providing hands-on dental treatment. While knowledge increased and uncertainty decreased during the course, the students’ general satisfaction was not assessed.

Our study design has methodological strengths and limitations. A major strength of our study is the combined quantitative and qualitative evaluation, allowing for validation of the satisfaction questionnaire by triangulation and for efficiently addressing reasons for low student satisfaction by modifying the course according to their free-text comments. Another strength arises from the prospective study design, enabling us to assess the effects of course modifications on student satisfaction. Furthermore, evaluations were anonymous, ensuring an unbiased evaluation without fear of consequences for their future studies or exams. Since both assessments were anonymous, the results had to be considered as two independent samples and not as repeated measures, which somewhat reduced the statistical power. Nonetheless, sample size and statistical power were sufficient, since the expected increase in student satisfaction scores was statistically significant. No control group without any modification of the course was available, as it was considered unethical to withhold the “best” level teaching from one group of students. Therefore, we cannot be sure that the effect on student satisfaction ratings was solely due to the course modification.

Time of exposure to elderly patients might have played a role, as ageism in students is known to diminish with increased exposure. Although ageism was not evaluated among students in the current study, it might have influenced the initial rating of the students with respect to satisfaction with the course. Prejudice and negative attitudes toward the aging process and confrontation with death are frequent among dental undergraduate students [10]. The proposed gerodontology course might have reduced ageism among the students [32], and evaluation of this attitude can be considered, as the course will be improved further during the follow-up of this study. Aging panels within undergraduate teaching have proven successful in fostering a positive attitude toward the elderly [33]. Dental students participating in geriatric ward rounds in an education project in Canada reported a more positive attitude towards domiciliary care after exposure to the LTC oral-care setting and the experience of providing oral health care to elderly, dependent patients [34]. They described the experience as “unique and emotionally challenging, but very worthwhile”. Elderly individuals living somewhat isolated in an LTC facility may particularly enjoy the visit of a young student examining their teeth and dentures. This dental visit may be a social highlight of their day, and rather than considering a dental professional as a service provider, they see the student as a social partner, and often share much more information with them then just their medical and dental anamnesis. The benefits of such “bonding” between oral health care providers and patients may not be limited to the provision of dental services, it may also foster the expansion of treatment options to less evidence-based approaches, as this increased trust enables sharing the risks of a possible failure. In the second module of the gerodontology course, the students may have experienced the agreeable exchange with the eldery and lost fear of the “unknown” situation. They may have felt more confident in performing gestures and treatments under non-ideal conditions, such as an unfavorable posture when the patient could not be transferred and inclined on the dental chair, poor visibility due to the lack of a performant lamp, some low-performance mobile equipment including limited suction of saliva and/or sudden movements of the patients, related to their underlying condition or medication.

Further reasons for the positive evaluations may relate to the fact that the course was not a mandatory element of their dental curriculum, so that the enrolled students were possibly more highly motivated and satisfied in this course compared to compulsory courses. The latter aspect should have affected the assessments of both modules similarly, and can therefore not account for the increase in satisfaction between semesters. One weakness of this study is that the response rate dropped from 92.3 to 70.0% from the first to the second evaluation, respectively; hence, the results do not represent all participants. However, since 50 of the initial 52 participants completed the second module, we assume that the low response rate was not due to dissatisfaction, but rather a lack of interest or time in completing the assessment form after the last module was finished, when at this moment they were free to engage in their desired leisure activities. At their young age, students have limited experience with conducting research projects and may therefore not fully understand the impact of dropping out on the validity of the study results. Looking positively at the dropout rate, it confirms the voluntary participation in the project. A final shortcoming of the study is that the course was limited to 1 year, preventing us from assessing long-term satisfaction, and the effect of the undergraduate training on future professional activities.

The newly developed gerodontology course was not subject to external review so far. However, since development was theoretically and practically driven by guidelines of the ECG, contents of German dental licensing regulations, previous experiences in a program on gerodontology, findings from a literature review on courses in gerodontology, and our interdisciplinary team including not only dentists but also specialists in general medicine, high quality of the course should be ensured. Furthermore, the principal investor is teaching gerodontology for several years. For development of the evaluation instrument, a sophisticated methodology was applied and the target population was included, ensuring validity of the instrument’s findings. This approach is similar to the development and validation of an instrument assessing student expectations of learning analytics services [35, 36].

The presently tested gerodontology course may serve as a prototype for other universities, since there is a general agreement that oral health care for the elderly is of increasing importance. Recent evidence regarding the associations between oral infections and general health, e.g. in cardiovascular disease, diabetes, osteoporosis, and institutionalized elders’ aspiration pneumonia, underline that oral health has a much larger impact than assumed some decades ago. Furthermore, recent dental research has increasingly included patient-reported outcomes [37], which evinced a close relation of oral health and oral-health related quality of life. Consequently, to prepare the dental health profession for upcoming public health challenges, gerodontology education should be reinforced not only on an undergraduate but also on a postgraduate level to strengthen key competencies of qualified dental professionals.

Furthermore, the applied combined quantitative and qualitative evaluation could be useful for the development of courses in general, because the approach is not specific for gerodontology. Further studies could benefit from the combined quantitative and qualitative evaluation for implementing a new course but also for increasing students’ satisfaction of existing courses.

## Conclusion

The combined quantitative and qualitative evaluation is a successful method for developing, implementing, evaluating, and improving a gerodontology course. Combining interdisciplinary theoretical education with practical experience in long-term care facilities and including students at various educational levels appears to improve the skills of future dentists with respect to providing oral health care to care-dependent elderly patients.

## Supplementary information

**Additional file 1.** Questionnaire. Evaluation of learning experience in a gerodontology course.

## Data Availability

The datasets generated and analyzed during the current study are available from the corresponding author on reasonable request.
