# Supplementary material for: Benefits of combined quantitative and qualitative evaluation of learning experience in a gerodontology course for dental students
Source: BMC Med Educ. 2020 Aug 26;20:281. doi: 10.1186/s12909-020-02196-0 (PMC7449035; doi:10.1186/s12909-020-02196-0)
Supplement: Supplementary file 1 — Additional file 1. Questionnaire. Evaluation of learning experience in a gerodontology course. [file 12909_2020_2196_MOESM1_ESM.pdf]

UKE HAMBURG
Center for Dental and Oral Medicine
Department of Prosthetic Dentistry

Evaluation of learning experience in a gerodontology course

	**Statement**	**not satisfied at all** … **very satisfied**
1	I am satisfied with the **time frame** of the course.	O O O O O O
2	I **do not** feel **emotionally overwhelmed** by the demands of the course.	O O O O O O
3	I am satisfied with the **implementation possibilities** within the course (e.g. permanent care of the same patient).	O O O O O O
4	I am satisfied with the **supervision** provided by the lecturers during the course.	O O O O O O
5	I experience a **cooperative collaboration** with other professional groups in the nursing homes.	O O O O O O
6	I make important **human experiences** within the course.	O O O O O O
7	I experience the **semester across collaboration** as enriching/helpful.	O O O O O O
8	I am satisfied with the **interdisciplinary education** (e.g. general medicine).	O O O O O O
9	By participating the course, I acquire **new knowledge** in dealing with older patients.	O O O O O O
10	By participating the course, I am **sensitized** to the needs of elderly patients.	O O O O O O
11	By participating the course, I am well **prepared for my professional life**.	O O O O O O
12	By participating the course, I improve my **communicative skills** in dealing with elderly patients.	O O O O O O
13	By participating the course, I gain new knowledge about the **effects of systemic diseases and oral health** in old age.	O O O O O O
14	By participating the course, I gain new knowledge about the **treatment options** for patients with geriatric diseases.	O O O O O O
15	By participating the course, I improve my **social competencies**.	O O O O O O
16	I am **generally satisfied** with the course.	O O O O O O
	**Praise:**<br><br>**Criticism:**<br><br>**Suggestions for improvement:**	
